# A prediction model for childhood obesity in New Zealand

**DOI:** 10.1038/s41598-021-85557-z

**Published:** 2021-03-18

**Authors:** Éadaoin M. Butler, Avinesh Pillai, Susan M. B. Morton, Blake M. Seers, Caroline G. Walker, Kien Ly, El-Shadan Tautolo, Marewa Glover, Rachael W. Taylor, Wayne S. Cutfield, José G. B. Derraik, COPABS Collaborators, Barbara C. Galland, Barry J. Taylor, Peter Tricker

**Affiliations:** 1 A Better Start-National Science Challenge, Auckland, New Zealand; 2 Liggins Institute, University of Auckland, Auckland, New Zealand; 3 Growing Up in New Zealand, Centre for Longitudinal Research, University of Auckland, Auckland, New Zealand; 4 Department of Statistics, University of Auckland, Auckland, New Zealand; 5 Department of Public Health and Psychosocial Studies, Auckland University of Technology, Auckland, New Zealand; 6 School of Public Health, College of Health, Massey University, Auckland, New Zealand; 7 Centre of Research Excellence Indigenous Sovereignty and Smoking, Auckland, New Zealand; 8 Department of Medicine, University of Otago, Dunedin, New Zealand; 9 Department of Women’s and Children’s Health, Uppsala University, Uppsala, Sweden; 10 Department of Endocrinology, Children’s Hospital, Zhejiang University School of Medicine, Hangzhou, China; 11 Department of Women’s and Children’s Health, University of Otago, Dunedin, New Zealand

**Keywords:** Obesity, Risk factors

## Abstract

Several early childhood obesity prediction models have been developed, but none for New Zealand's diverse population. We aimed to develop and validate a model for predicting obesity in 4–5-year-old New Zealand children, using parental and infant data from the Growing Up in New Zealand (GUiNZ) cohort. Obesity was defined as body mass index (BMI) for age and sex ≥ 95th percentile. Data on GUiNZ children were used for derivation (n = 1731) and internal validation (n = 713). External validation was performed using data from the Prevention of Overweight in Infancy Study (POI, n = 383) and Pacific Islands Families Study (PIF, n = 135) cohorts. The final model included: birth weight, maternal smoking during pregnancy, maternal pre-pregnancy BMI, paternal BMI, and infant weight gain. Discrimination accuracy was adequate [AUROC = 0.74 (0.71–0.77)], remained so when validated internally [AUROC = 0.73 (0.68–0.78)] and externally on PIF [AUROC = 0.74 [0.66–0.82)] and POI [AUROC = 0.80 (0.71–0.90)]. Positive predictive values were variable but low across the risk threshold range (GUiNZ derivation 19–54%; GUiNZ validation 19–48%; and POI 8–24%), although more consistent in the PIF cohort (52–61%), all indicating high rates of false positives. Although this early childhood obesity prediction model could inform early obesity prevention, high rates of false positives might create unwarranted anxiety for families.

## Introduction

Worldwide, an estimated 38 million children under the age of 5 years are above the body mass index (BMI) cut-off for what is considered a healthy weight^1^. In New Zealand, 14.9% of 4–5-year-olds have obesity, and 2.9% have extreme obesity^2^, with Māori and Pacific children experiencing disproportionately higher levels of obesity than children from other ethnic groups^2^. Childhood obesity tracks into later life^3^, even in the very young; having a body mass index (BMI) or weight for length at or above the World Health Organization (WHO) 85th percentile before the age of 18 months predicts obesity at 6 years of age^4^. Such findings highlight the importance of early intervention.

There are a number of potentially modifiable risk factors associated with early childhood obesity, including maternal smoking during pregnancy^5–7^, high maternal pre-pregnancy BMI^5–7^, excessive gestational weight gain^5,6^, high birth weight^5,7^, rapid infant weight gain^5,7^, high-protein infant formula^7^, and poor infant sleep^5,7^. Despite the identification of potentially modifiable risk factors, early childhood obesity interventions have reported inconsistent results^8^, although a recent meta-analysis of four trials, including one based in New Zealand, reported that early intervention reduced infants’ BMI *z-*scores by 18–24 months^9^. However, health professionals report they lack the knowledge required to confidently identify obesity risk in young children^10–13^. Providing health professionals with a tool that enables accurate prediction of an infant’s risk of obesity could serve to increase the effectiveness of early childhood obesity interventions, through enabling timely intervention. Importantly, any such model would need to be accurate enough to warrant telling families what could be worrying information for them^14^. In particular, a prediction model with a low positive predictive value (PPV, i.e. the probability that those considered to be at risk by the model will actually go on to develop obesity) would likely create considerable unwarranted anxiety.

Internationally, several prediction models have been developed to determine risk of early childhood obesity^15^, but to date no such model has been developed for the ethnically diverse New Zealand population. Prediction models should be developed and validated using participant data that are representative of the population in which the model will be used^16^. Thus, we aimed to develop, internally validate, and externally validate a prediction model for obesity at 4–5-years-of-age for New Zealand children. Additionally, because the prevalence of severe childhood obesity is of increasing concern, and children with severe obesity tend to have poorer clinical outcomes compared to those with less severe forms^17^, we also aimed to derive and validate models for severe childhood obesity in the same population.

## Methods

### Primary study population

The derivation and internal validation samples were obtained using data from the Growing Up in New Zealand (GUiNZ) study, a longitudinal, prospective birth cohort that recruited 6853 children via their pregnant mothers with an estimated due date between 25 April 2009 and 25 March 2010, who were resident in the greater Auckland, Counties-Manukau, and Waikato District Health Boards regions of New Zealand^18^. The cohort characteristics and recruitment strategy have been described elsewhere^18^. The generalizability of the recruited cohort to all current New Zealand births has also been demonstrated, especially with regard to ethnicity and socio-demographics^19^. Multiple data collection waves have occurred from pregnancy and throughout the pre-school period. Sociodemographic information including parental education, ethnicity, and socioeconomic deprivation were collected during pregnancy, as was maternal pre-pregnancy BMI and paternal BMI. Consent for linkage to routine birth records was also sought from parents to complement parental reported size at birth measures^18^. Socioeconomic status was determined using the NZDep2006^18,20^, an area level measure of deprivation, which provides scores ranging from 1 (least deprived areas) to 10 (most deprived areas)^20^. At ages 2 and 4 years, trained GUiNZ researchers obtained height and weight measurements from the children using standardised protocols during face-to-face home interviews^21^.

### Populations for external validation

Two populations were used for external validation of the prediction models. External validation is vital to determine the generalisability of a prediction model to populations that are reflective of, but not identical to, the population used to derive the model^22^. Prevention of Overweight in Infancy (POI) was a randomised controlled trial of interventions to prevent overweight during infancy^23^. In brief, 802 mothers and infants living in Dunedin (New Zealand) were recruited between 2009 and 2012. Pregnant women were eligible to participate if they were over 16 years of age, could communicate in English or the indigenous Māori language, and were planning to live in the Dunedin area for the following 2 years^23^. Anthropometric measurements were taken by trained researchers when the children were 5 years old^24^. The majority of children in POI (79%) were of New Zealand European ethnicity^23^.

The Pacific Islands Families (PIF) study recruited newborn infants of Pacific Island ethnicity born at Middlemore Hospital (Auckland, New Zealand) between March and December 2000^25^. Infants were considered to be of Pacific Island ethnicity if at least one parent identified themselves as such. Participants joined the study at their 6-week follow-up, when 1376 eligible mothers (1398 infants) consented to and completed an interview. Children’s heights and weights were measured by trained researchers using standardized equipment at approximately 4 years of age^26^.

### Outcome

Children's BMI measures were transformed into *z*-scores as per WHO standards^28,29^. The primary outcome for the childhood obesity model was a binary outcome of obesity at age 4–5 years, which was defined as a BMI *z*-score ≥ 1.645 (or ≥ 95th percentile for age and sex)^30^.

A further two models were derived to predict severe childhood obesity. The outcome for the first was the likelihood of severe childhood obesity at age 4–5 years, defined as a BMI *z-*score ≥ 1.974 (i.e. ≥ 120% of the 95th percentile for age and sex); for the second model, it was defined as a BMI *z*-score ≥ 2.326 (or ≥ 99th percentile for age and sex)^17^.

### Model development

Participants from GUiNZ with complete maternal and paternal data were randomly split into derivation (70%) and validation (30%) cohorts. A large number of categorical and continuous variables were selected from GUiNZ (Supplementary Table S1). The associations between categorical variables and obesity in childhood were examined using Chi-square tests. For continuous variables, univariable logistic regressions were used. All parameters displaying an association with p < 0.10 were selected for the next step.

Pairwise associations between all continuous variables were examined using Pearson's correlation coefficients, with high collinearity defined as |r|≥ 0.5^31^. Similarly, associations between categorical variables were also examined to identify cases of high collinearity based on the phi (φ) coefficient: |φ|≥ 0.5. Whenever high collinearity between two variables was identified, one of these variables was eliminated, primarily based on their practicality for use in routine clinical practice. The remaining parameters were selected for further investigation. In addition, two-way interactions between all remaining variables were examined, and those that were statistically significant at p < 0.05 were also selected.

Ethnicity in GUiNZ was self-reported, and participants were able to select multiple ethnic groups with which they identified, and to self-prioritise their main ethnicity. Children’s expected ethnicity was also reported by their mothers^19^, but for a number of reasons, maternal prioritised ethnicity was used to represent children’s ethnicity in this study. Firstly, paternal demographic information is not always reliably available during pregnancy. Secondly, previous work on GUiNZ data has demonstrated that children’s reported ethnicity can vary by parent^32^. Lastly, maternal contributions to overweight or obesity in offspring, or risk factors such as high birth-weight, have been shown to be equal to^33,34^ or greater than^35,36^ that of paternal contributions. Ethnicity was defined using a hierarchical system of classification, such that if multiple ethnicities were selected, the participant was assigned to a single category^37^. Given the small numbers of participants in the categories ‘Other’ and ‘MELAA’ (Middle Eastern, Latin American and African), these were combined as ‘Other ethnicities’.

Model discrimination was defined as its ability to adequately differentiate those with high risk of an event from those with a low risk^38^, and it was estimated using the area under the receiver operating characteristic curve (AUROC). The following thresholds to assess model discrimination based on AUROC were adopted: poor (< 0.60), possibly useful (≥ 0.60 and < 0.70), acceptable (≥ 0.70, < 0.80), excellent (≥ 0.80 and < 0.90), and outstanding (≥ 0.90)^38,39^.

Model calibration was defined as its goodness of fit or extent to which the model correctly estimated the absolute risk^38^, and was determined using the Hosmer–Lemeshow test^39^. A p value < 0.05 was considered evidence of poor model calibration.

In addition, the following parameters were obtained to assess model accuracy and predictive capacity:

$$Sensitivity=\frac{\mathrm{n} \, \mathrm{true} \, \mathrm{positives }}{(\mathrm{n} \, \mathrm{true} \, \mathrm{positives}+\mathrm{ n} \, \mathrm{ false} \, \mathrm{negatives})}$$

$$Specificity=\frac{\mathrm{n} \, \mathrm{true} \, \mathrm{negatives}}{(\mathrm{n} \, \mathrm{true} \, \mathrm{negatives}+\mathrm{ n} \, \mathrm{false} \, \mathrm{positives})}$$

$$Positive \, predictive \, value \, (PPV)=\frac{\mathrm{n} \, \mathrm{true} \, \mathrm{positives }}{\left(\mathrm{n} \, \mathrm{true} \, \mathrm{positives }+\mathrm{ n} \, \mathrm{false} \, \mathrm{positives}\right)}$$

$$Negative \, predictive \, value \, (NPV)=\frac{\mathrm{n} \, \mathrm{true} \, \mathrm{negatives }}{(\mathrm{n} \, \mathrm{true} \, \mathrm{negatives }+\mathrm{ n} \, \mathrm{false} \, \mathrm{negatives})}$$

Using the derivation cohort, selected variables were included in a logistic regression model. Models were progressively modified with the addition and/or removal of parameters. This step was repeated multiple times, with model discrimination and calibration assessed at each step, until the most parsimonious model with the best discrimination possible was obtained, while accounting for a predictor's usability in routine practice. Using the method described by Moons et al.^22^, the model was then internally and externally validated, with the same equation applied to the GUiNZ internal validation cohort, and POI and PIF external validation cohorts.

Analyses were performed in SPSS v25 (IBM Corp, Armonk, NY, USA) and SAS v9.4 (SAS Institute, Cary, USA). All tests were two-tailed, with statistical significance set at p < 0.05 and without adjustment for multiple comparisons. There was no imputation of missing data.

### Ethics approval

This study solely involved the use of anonymized data. Ethics approval for GUiNZ was granted by the Ministry of Health Northern Y Regional Ethics Committee, New Zealand^18^; for POI by the New Zealand Lower South Regional Ethics Committee, New Zealand^27^; and for PIF by the Auckland branch of the National Ethics Committee, New Zealand^26^. This study was performed in accordance with all appropriate institutional and international guidelines and regulations for research. Written informed consent was obtained from a parent or legal guardian of all participants from the individual studies.

### Disclosure

The authors have no financial or non-financial conflicts of interest to disclose that may be relevant to this work. The funders had no role in study design, data analysis or interpretation, decision to publish, or preparation of this manuscript.

## Results

### GUiNZ

The selected derivation cohort consisted of 1731 participants, while the selected internal validation cohort consisted of 713 participants (Supplementary Figure S1). The number of participants that we ultimately used was reduced mostly due to the lack of paternal and/or infancy weight gain data (Supplementary Figure S1), which were identified as key parameters during variable selection. Of note, Supplementary Table S2 shows that there were some differences between included and excluded GUiNZ participants. In particular, the maternal ethnicity and sociodemographic status of participants differed, with more participants with mothers of Māori or Pacific ethnicity and/or from areas of greater socioeconomic deprivation in the excluded group. The exclusion of Māori and Pacific mother-infant pairs, primarily due to missing paternal data, is likely to have introduced some bias. However, ethnic-specific models (New Zealand European, Māori, and Pacific) were also derived, and yielded no clear advantage in comparison to the overall model (data not shown). For those included participants, the prevalence of obesity was similar in the GUiNZ derivation and validation cohorts (15.8% and 16.1%, respectively), while other demographic factors were also similar (Table 1).

**Table 1:** Demographic information on the populations used for model derivation and validation.

	GUiNZ derivation	GUiNZ validation	POI	PIF
**n**	1731	713	383	135
**Age (years)**	4.5 ± 0.1	4.5 ± 0.1	5.0 ± 0.0	4.1 ± 0.1
**Sex (males)**	912 (52.7%)	377 (52.9%)	193 (50.4%)	76 (56.3%)
**Birth weight (kg)**	3.47 ± 0.56	3.49 ± 0.58	3.59 ± 0.47	3.69 ± 0.56
**Birth weight z-score**	0.68 ± 1.00	0.70 ± 1.01	0.60 ± 0.97	0.63 ± 1.06
**BMI z-score**	0.75 ± 1.06	0.78 ± 0.95	0.44 ± 0.88	1.88 ± 1.45
**Child BMI status**				
Underweight/normal weight	1111 (64.2%)	446 (62.6%)	298 (77.8%)	34 (25.2%)
Overweight	346 (20.0%)	152 (21.3%)	58 (15.1%)	32 (23.7%)
Obesity	274 (15.8%)	115 (16.1%)	27 (7.0%)	69 (51.1%)
**Number of other persons in household**	2 [2, 3]	2 [2, 3]	3 [2, 3]	4 [3, 6]
**Maternal ethnicity**				
New Zealand European	1236 (71.5%)	489 (68.6%)	346 (90.3%)	–
Māori	111 (6.4%)	59 (8.3%)	13 (3.4%)	–
Pacific	89 (5.2%)	30 (4.2%)	1 (0.3%)	–
Asian	252 (14.6%)	114 (16.0%)	15 (3.9%)	–
Other	40 (2.3%)	21 (2.9%)	8 (2.1%)	–
**Maternal smoking during pregnancy**	78 (4.5%)	35 (4.9%)	20 (5.2%)	25 (18.5%)
**Maternal education**				
High-school or lesser	343 (19.8%)	136 (19.1%)	48 (12.5%)	93 (68.9%)
Post-secondary	451 (26.1%)	212 (29.7%)	50 (13.1%)	36 (26.7%)
University	936 (54.1%)	365 (51.2%)	285 (74.4%)	6 (4.4%)
**NZDep2006**				
1 (least deprived)	364 (21.0%)	169 (23.7%)	95 (25.5%)	–
2	402 (23.2%)	147 (20.6%)	99 (26.3%)	–
3	351 (20.3%)	132 (18.5%)	89 (23.6%)	–
4	337 (19.5%)	151 (21.2%)	77 (20.4%)	–
5 (most deprived)	277 (16.0%)	113 (15.9%)	17 (4.5%)	–
**Mother's pre-pregnancy BMI (kg/m**^**2**^**)**	24.91 ± 5.33	24.86 ± 5.30	24.84 ± 4.84	37.26 ± 8.25
**Mother's pre-pregnancy BMI status**				
Underweight/normal weight	1080 (62.4%)	438 (61.4%)	245 (64.0%)	9 (6.7%)
Overweight	383 (22.1%)	159 (22.3%)	94 (24.5%)	13 (9.6%)
Obesity	268 (15.5%)	116 (16.3%)	44 (11.5%)	113 (83.7%)
**Father's BMI (kg/m**^2^**)**	27.29 ± 4.77	27.27 ± 4.58	27.09 ± 4.30	36.73 ± 6.59
**Father's BMI status**				
Underweight/normal weight	585 (33.8%)	242 (33.9%)	131 (34.2%)	2 (1.5%)
Overweight	762 (44.0%)	323 (45.3%)	171 (44.6%)	13 (9.6%)
Obesity	384 (22.2%)	148 (20.8%)	81 (21.1%)	120 (88.9%)

*BMI* body mass index, *GUiNZ* Growing Up in New Zealand Study, *NZDep2006* New Zealand Index of [socioeconomic] Deprivation 2006, *PIF* Pacific Islands Families Study, *POI* Prevention of Overweight in Infancy Study.

Where appropriate, data are n (%) or means ± standard deviations (SD), except for "Number of other persons in household" whose data are medians [quartile 1, quartile 3].

BMI status for children: underweight/normal weight BMI *z*-score < 1.036; overweight BMI *z*-score ≥ 1.036 and < 1.645; and obesity BMI *z*-score ≥ 1.645.

BMI status for mothers and fathers: underweight/normal weight BMI < 25 kg/m^2^; overweight ≥ 25 kg/m^2^ and < 30 kg/m^2^; and obesity ≥ 30 kg/m^2^.

### Model predictors

The final prediction model included maternal pre-pregnancy BMI, paternal BMI, birth weight, maternal smoking during pregnancy, and infant weight gain as significant independent predictors of childhood obesity (Table 2).

**Table 2:** Parameters for the final childhood obesity prediction model derived from the Growing Up in New Zealand subset.

	β	SEM	p value	Odds ratio (95% CI)
Birth weight (kg)	1.337	0.161	< 0.0001	3.81 (2.78, 5.22)
**Maternal pre-pregnancy BMI (kg/m**^**2**^**)**	0.050	0.012	< 0.0001	1.05 (1.03, 1.08)
**Paternal BMI (kg/m**^**2**^**)**^a^	0.063	0.014	< 0.0001	1.07 (1.04, 1.09)
**Monthly weight gain from birth (per 0.1** ***z*-score)**^**b**^	0.369	0.051	< 0.0001	1.45 (1.31, 1.60)
**Maternal smoking during pregnancy**	0.700	0.285	0.014	2.01 (1.15, 3.52)
**Constant**	− 9.492	0.722	< 0.0001	–

*BMI* body mass index, *CI* confidence interval, *SEM* standard error of the mean.

^a^Collected during partner’s pregnancy.

^b^Average monthly change in weight *z*-score from birth until the last recorded measurement between 6 and 12 months of age.

### Derivation

Discrimination accuracy was acceptable for the derivation model [AUROC = 0.74 (0.71, 0.77), p < 0.0001], and there was no evidence of poor model calibration (p value for Hosmer–Lemeshow test = 0.27). Table 3 shows the sensitivity, specificity, PPV, and NPV of the derivation model at various risk thresholds ranging from 0.20 to 0.95. PPVs ranged from 18.6 to 54.0% (Table 3).

**Table 3:** Accuracy and predictive capacity of a prediction model for childhood obesity for New Zealand among the derivation and validation cohorts.

Model	Parameter	Probability percentile threshold															
		20	25	30	35	40	45	50	55	60	65	70	75	80	85	90	95
GUiNZ derivation model	True positives (n)	257	251	246	240	232	224	214	202	192	183	166	146	133	107	80	47
n = 1731	True negatives (n)	329	410	491	572	650	729	805	880	956	1034	1103	1170	1243	1304	1363	1417
AUROC = 0.74 (0.71, 0.77)	False positives (n)	1128	1047	966	885	807	728	652	577	501	423	354	287	214	153	94	40
	False negatives (n)	17	23	28	34	42	50	60	72	82	91	108	128	141	167	194	227
	Sensitivity (%)	93.8	91.6	89.8	87.6	84.7	81.8	78.1	73.7	70.1	66.8	60.6	53.3	48.5	39.1	29.2	17.2
	Specificity (%)	22.6	28.1	33.7	39.3	44.6	50.0	55.3	60.4	65.6	71.0	75.7	80.3	85.3	89.5	93.5	97.3
	PPV (%)	18.6	19.3	20.3	21.3	22.3	23.5	24.7	25.9	27.7	30.2	31.9	33.7	38.3	41.2	46.0	54.0
	NPV (%)	95.1	94.7	94.6	94.4	93.9	93.6	93.1	92.4	92.1	91.9	91.1	90.1	89.8	88.6	87.5	86.2
GUiNZ internal validation model	True positives (n)	107	105	103	100	97	92	86	82	80	74	65	59	53	48	42	21
n = 713	True negatives (n)	143	176	222	243	270	293	332	360	383	413	438	469	499	527	550	575
AUROC = 0.73 (0.68, 0.78)	False positives (n)	455	422	376	355	328	305	266	238	215	185	160	129	99	71	48	23
	False negatives (n)	8	10	12	15	18	23	29	33	35	41	50	56	62	67	73	94
	Sensitivity (%)	93.0	91.3	89.6	87.0	84.3	80.0	74.8	71.3	69.6	64.3	56.5	51.3	46.1	41.7	36.5	18.3
	Specificity (%)	23.9	29.4	37.1	40.6	45.2	49.0	55.5	60.2	64.0	68.9	73.2	78.4	83.4	88.1	92.0	96.2
	PPV (%)	19.0	19.9	21.5	22.0	22.8	23.2	24.4	25.6	27.1	28.6	28.9	31.4	34.9	40.3	46.7	47.7
	NPV (%)	94.7	94.6	94.9	94.2	93.8	92.7	92.0	91.6	91.6	91.0	89.8	89.3	88.9	88.7	88.3	85.9
POI external validation model	True positives (n)	27	26	26	26	26	26	24	23	21	21	20	17	17	12	5	4
n = 383	True negatives (n)	37	50	80	97	114	136	167	182	210	236	257	276	291	309	325	343
AUROC = 0.74 (0.66, 0.82)	False positives (n)	319	306	276	259	242	220	189	174	146	120	99	80	65	47	31	13
	False negatives (n)	0	1	1	1	1	1	3	4	6	6	7	10	10	15	22	23
	Sensitivity (%)	100	96.3	96.3	96.3	96.3	96.3	88.9	85.2	77.8	77.8	74.1	63.0	63.0	44.4	18.5	14.8
	Specificity (%)	10.4	14.0	22.5	27.2	32.0	38.2	46.9	51.1	59.0	66.3	72.2	77.5	81.7	86.8	91.3	96.3
	PPV (%)	7.8	7.8	8.6	9.1	9.7	10.6	11.3	11.7	12.6	14.9	16.8	17.5	20.7	20.3	13.9	23.5
	NPV (%)	100	98.0	98.8	99.0	99.1	99.3	98.2	97.8	97.2	97.5	97.3	96.5	96.7	95.4	93.7	93.7
PIF external validation model	True positives (n)	69	69	69	69	69	69	68	68	68	67	67	65	65	64	60	52
n = 135	True negatives (n)	2	2	2	3	4	6	6	6	8	10	12	12	14	17	25	32
AUROC = 0.80 (0.71, 0.90)	False positives (n)	64	64	64	63	62	60	60	60	58	56	54	54	52	49	41	34
	False negatives (n)	0	0	0	0	0	0	1	1	1	2	2	4	4	5	9	17
	Sensitivity (%)	100	100	100	100	100	100	98.6	98.6	98.6	97.1	97.1	94.2	94.2	92.8	87.0	75.4
	Specificity (%)	3.0	3.0	3.0	4.5	6.1	9.1	9.1	9.1	12.1	15.2	18.2	18.2	21.2	25.8	37.9	48.5
	PPV (%)	51.9	51.9	51.9	52.3	52.7	53.5	53.1	53.1	54.0	54.5	55.4	54.6	55.6	56.6	59.4	60.5
	NPV (%)	100	100	100	100	100	100	85.7	85.7	88.9	83.3	85.7	75.0	77.8	77.3	73.5	65.3

*AUROC* area under the receiver operating characteristic curve, *GUiNZ* Growing Up in New Zealand Study, *NPV* negative predictive value, *PIF* Pacific Islands Families Study, *POI* Prevention of Overweight in Infancy Study, *PPV* positive predictive value.

### Internal validation

Discrimination accuracy was acceptable for the internal validation model [AUROC = 0.73 (0.68, 0.78), p < 0.0001]. There was no evidence of poor model calibration (p for Hosmer–Lemeshow test = 0.75). Model accuracy parameters are shown in Table 3. Similar to the derivation model, PPVs ranged from 19.0 to 47.7% (Table 3).

### External validation

The demographic information for each of the external validation cohorts is provided in Table 1. Notably, the prevalence of childhood obesity in the POI cohort was 7.0% and 51.1% in PIF (Table 1).

Discrimination accuracy was excellent for the POI cohort [AUROC = 0.80 (0.71, 0.90), p < 0.0001] and acceptable for PIF [AUROC = 0.74 (0.66, 0.82), p < 0.0001]. There was no evidence of poor model calibration (all p > 0.05 for Hosmer–Lemeshow test).

Table 3 provides the sensitivity, specificity, PPV, and NPV of the external validation models at various percentile risk thresholds ranging from 0.20 to 0.95. PPVs were particularly low for POI, ranging from 7.8 to 23.5%, but higher and more consistent for PIF, ranging from 51.9 to 60.5% (Table 3).

### Severe childhood obesity prediction

Both models with severe childhood obesity as the outcome included 2408 participants in their derivation cohorts (Supplementary Table S3). The predictors included for each model and their associations with the outcome are reported in Supplementary Tables S4 and S5. Discrimination accuracy was acceptable for the model predicting severe childhood obesity at BMI ≥ 120% of the 95th percentile [AUROC = 0.75 (0.72, 0.78); Supplementary Table S6] and at BMI ≥ 99th percentile [AUROC = 0.76 (0.72, 0.80); Supplementary Table S7]. There was no evidence of poor calibration for either derivation model (both p > 0.05 for Hosmer–Lemeshow test) or for the validation models (all p > 0.05 also), with the exception of the POI validation model for severe childhood obesity at a BMI *z-*score ≥ 99th percentile (p = 0.002).

The sensitivity, specificity, PPV, and NPV of the derivation and validation models for severe childhood obesity are reported in Supplementary Tables S6 and S7. PPVs for both derivation models were low, ranging from 15.0 to 47.9% for the model predicting severe childhood obesity at BMI ≥ 120% of the 95th percentile (Supplementary Table S6), and 9.0 to 33.1% at BMI ≥ 99th percentile (Supplementary Table S7). The PPVs for the internal validation model predicting severe childhood obesity at BMI ≥ 120% of the 95th percentile were similarly low to those of the derivation model (14.2–51.0%, Supplementary Table S6), as were the PPVs for the model at BMI ≥ 99th percentile (7.8–34.5%, Supplementary Table S7). Regarding external validations, the PPVs for both POI models were particularly poor (5.2–16.7%, Supplementary Table S6; 0–8.7%, Supplementary Table S7), although markedly higher for the PIF models (38.3–55.5%, Supplementary Table S6; 41.5–66.7%, Supplementary Table S7).

## Discussion

New Zealand has a high prevalence of early childhood obesity, with one in three children starting school already overweight or with obesity^2^. Furthermore, New Zealand’s population is an ethnically diverse one^40^, with obesity rates inequitably distributed across ethnicities. For example, 30.2% of Pacific Island children have obesity by the age of four, versus 12.7% of New Zealand European children^2^. We have developed a model to attempt to predict likelihood of obesity at 4–5 years based on infancy data, starting with a cohort that is representative of the New Zealand birth population^19^. The derivation model, internal validation model, and two external validation models all produced AUROCs that were either acceptable or excellent. However, despite the encouraging AUROCs, PPVs were almost invariably low across the risk threshold range, indicating high rates of false positives. In addition, derivation and validation of two models to predict severe childhood obesity produced similar findings.

The performance of our childhood obesity prediction model was comparable with previous international early childhood obesity or overweight prediction models, with derivation AUROCs ranging from 0.67 to 0.87^15^. Our final model used five predictors: birth weight, maternal pre-pregnancy BMI, paternal BMI, infancy weight gain, and maternal smoking during pregnancy. The inclusion of infancy weight gain data in early childhood obesity prediction regression models has been suggested to produce higher AUROC values than models solely reliant on predictors available at birth^15^. Our model produced a higher AUROC than one previous regression model supplemented by infancy weight gain data, but was lower than two others^15^. However, our infancy weight gain data were obtained using only two time points: birth and anywhere between 6 and 12 months. It is possible that data collected at more regular intervals would have improved our model’s discrimination.

Our model’s discrimination capacity was excellent for the POI cohort, while it remained acceptable for the PIF cohort. This may be explained by differences between the populations. POI was a cohort consisting of mostly New Zealand Europeans, with a low prevalence of obesity at 7.0%. By comparison, the PIF cohort included only Pacific Island children, with a markedly higher prevalence of obesity at 51.1%. However, of particular relevance, there were also substantial contrasts in PPVs when the model was applied to these external validation cohorts. For example, the highest PPV achieved when the model was applied to the POI cohort was 23.5% compared to 60.5% in the PIF cohort. In actual numbers, at the 95th probability percentile threshold (where the PPV was highest), 52 of 69 participants in the PIF cohort who did develop obesity were correctly identified as being likely to do so (sensitivity = 75.4%), while 34 were incorrectly identified as at risk. By contrast, at the same threshold, the model correctly identified only 4 of 27 participants that went on to develop obesity in the POI cohort (sensitivity = 14.8%), with 13 false positives. Choosing a lower risk threshold would only serve to increase the rate of false positives. Of note, derivation and validation of models to predict severe childhood obesity according to two definitions (known to be associated with poorer long-term clinical outcomes compared to less severe obesity^17^) did not substantially improve PPVs for any included cohort.

Risk thresholds should be determined following consideration of a number of factors, including sensitivity and specificity of the model, financial cost of any proposed intervention, and any potential risks of intervening or not intervening in misclassified cases^15^. For obvious reasons, the weight given to these individual aspects will vary markedly depending on the condition in question, whether it is a relatively benign condition vs a disease with a very high mortality rate (e.g. pancreatic cancer). In this study, the observed rates of false positives across the risk threshold continuum indicate that in New Zealand’s multi-ethnic population, a large number of children would be incorrectly classified as at risk of childhood obesity. There is a large social stigma associated with childhood obesity^41^, and a high number of false positives (i.e. children being wrongly labelled as at risk of developing obesity) could create considerable unwarranted anxiety for many families, and thus should be avoided. In addition, screening for obesity may be unethical in the absence of proven treatments for early childhood obesity. Nonetheless, a recent meta-analysis reported that randomised controlled trials on interventions to prevent early childhood obesity conducted in Australia and New Zealand (n=4) resulted in lower BMI *z-*scores at 18–24 months^9^.

There has been some research into the acceptability of early childhood obesity prediction to families. In New Zealand, parents and other caregivers were hypothetically accepting of communication regarding early childhood obesity prediction, although many anticipated feeling upset or worried in response^14^. UK parents were also hypothetically accepting of such communication, but were fearful of judgement from health professionals and others^42^. One study has explored parental views of actual risk communication^43^, reporting that some parents did not trust the prediction and believed the risk was not likely to be relevant for their baby^43^. Of note, some of the health professionals delivering the risk communication also expressed similar beliefs^43^. Indeed, parental awareness of current childhood obesity in older children is not necessarily associated with action to reduce the child’s weight^44^. Furthermore, there is some evidence that in the long-term, young children considered to have overweight by their parent gain more weight than children not considered to have overweight^45–47^. In one study, this was regardless of the child’s initial weight status (i.e. normal weight or overweight)^45^. Therefore, accurate parental perception of children’s weight status could even be detrimental to children’s long-term weight outcomes. Nonetheless, it is important to note that all three of these studies were observational in design, and no intervention was offered to children considered to have overweight (as would be the case if a prediction model like ours were to be used in clinical practice). Furthermore, a prediction model does not require parents to perceive their child as having a current weight issue, but rather to consider them at risk of developing obesity in the future. Therefore, use of an early childhood obesity prediction model could have different long-term outcomes to the previously mentioned longitudinal studies. A randomised controlled trial on the use of a prediction model (with lower false positive rates than ours), alongside supportive intervention, would be needed to assess if this was the case.

Our study has several limitations. Parental anthropometric and infancy weight gain data were missing for many participants in all cohorts, meaning these otherwise suitable participants were excluded from the final analysis. While the overall GUiNZ cohort was representative of New Zealand's population^19^, the exclusion of participants with missing data (in particular missing paternal data) meant that a greater proportion of children from lower socioeconomic backgrounds and/or with mothers of Māori or Pacific ethnicity, were excluded. Thus, this exclusion of participants should be considered when interpreting the applicability of the model to the wider New Zealand population. Also, parental BMI was based on self-reported data and potentially inaccurate^48^, although a New Zealand study found no differences between self-reported and measured BMI in adults^49^. In addition, our model was derived using historical data that might not be reflective of current trends. Nonetheless, all GUiNZ infants were born between 2009 and 2010^18^; the prevalence of obesity in 4–5-year-old New Zealand children has not increased since 2010 (regardless of ethnicity or socioeconomic status)^2^. Furthermore, the prevalence of obesity among New Zealand adults has been stable since 2012/13, with only a slight rise since 2011/12^50^. Taken together, this suggests that the prevalence of obesity among children included in our derivation cohort is likely to be reflective of current early childhood obesity trends in New Zealand children, and at least two of the models’ parameters (maternal pre-pregnancy BMI and paternal BMI) are also likely to be closely reflective of current trends. Additionally, while BMI ≥ 95th percentile is a commonly used threshold for obesity in children, it is acknowledged that BMI does not directly measure body fat^30^. Thus, it cannot be considered a definitive diagnostic tool for obesity, which is defined by the WHO to be “abnormal or excessive fat accumulation that may impair health”^51^. Nonetheless, its practicality means it is a commonly used tool for diagnosis of obesity in clinical practice^30^; in New Zealand for example, BMI (*z*-score) is a key tool to identify children with weight issues in the Ministry of Health's B4 School Check, a nationwide programme that offers free health and development assessments for all children at 4 years of age^52^. Strengths of our study include the internal and external validations, and all children’s anthropometric measurements being taken using consistent protocols by trained professionals, which reduces the potential for error.

In conclusion, we have derived and validated an early childhood obesity prediction model using a cohort that is representative of the diversity of the contemporary New Zealand birth population, although children of Māori and Pacific mothers and those from lower socioeconomic backgrounds were under-represented in our selected subset. While AUROCs for the derivation and validation models were at least acceptable, PPVs were generally low so that high rates of false positives could create considerable unwarranted anxiety for many families. This would be a particular issue in a context where there remains uncertainty regarding what constitutes a successful intervention to prevent early childhood obesity, thus, we are hesitant to recommend the use our model in clinical practice.

## Supplementary Information

Supplementary Information

## Data Availability

The study data cannot be made available in a public repository, due to the strict conditions of the ethics approvals of the original studies from which our data were derived. However, the anonymised data on which this manuscript was based could be made available to other investigators upon bona fide request, and following all the necessary approvals (including ethics) of the detailed study proposal and statistical analyses plan. Any queries should be directed to Dr José Derraik (j.derraik@auckland.ac.nz).
