# Supplementary material for: A prediction model for childhood obesity in New Zealand
Source: Sci Rep. 2021 Mar 18;11:6380. doi: 10.1038/s41598-021-85557-z (PMC7973754; doi:10.1038/s41598-021-85557-z)

Supplementary Information

Title: A prediction model for childhood obesity in New Zealand

Authors: Éadaoin M Butler, Avinesh Pillai, Susan M B Morton, Blake M Seers, Caroline G Walker, Kien Ly, El-Shadan Tautolo, Marewa Glover, Rachael W Taylor, Wayne S Cutfield, José G B Derraik, COPABS Collaborators

Supplementary Table 1

Initial list of variables for the Growing Up in New Zealand cohort that were considered for model derivation.

Type	Grouping	Description	Levels
**Categorical**	**Pregnancy**	Alcohol consumption in 1st trimester of pregnancy (per week)	0=None or less than 1 drink; 1=More than 1 drink
		Alcohol consumption after 1st trimester of pregnancy (per week)	0=None or less than 1 drink; 1=More than 1 drink
		Alcohol consumption in pregnancy	0=No alcohol; 1=Drank alcohol
		Hyperemesis during this pregnancy	0=None; 1=Hyperemesis
		Labour was induced	0=Not induced; 1=Induced
		Maternal hypertension during pregnancy	0=No; 1=Hypertension
		Mode of delivery	1=Vaginal; 2=C-section
		Mother living with the biological father at the time of childbirth	0=No; 1=Yes
		Maternal smoking during pregnancy	0=No; 1=Yes
		Partner smoking during pregnancy	0=No; 1=Yes
		Pre-eclampsia during pregnancy	0=No; 1=Yes
		Type of pregnancy (number of fetuses)	1=Singleton; 2=Twin/multiple
	**Mother**	Maternal diabetes	0=No; 1=Diabetes
		Mother's employment status at baseline	0=Unemployed; 1=Employed
		Mother's highest education as binary outcome	1=High-school or lesser; 2= Post-secondary
		Mother diagnosed with cardiovascular disease (CVD)	0=Never had CVD; 1=CVD before and/or during pregnancy
		Mother has a university degree	1=No; 2=Yes
		Mother's self-reported ethnicity	1=NZ European; 2=Māori; 3=Pacific; 4=Asian; 5=Other
		Parity (beyond 24 weeks of gestation)	0=Nulliparous; 1=Multiparous
	**Father**	Father's cardiovascular disease status	0=None; 1=CVD
		Father's diabetes status	0=None; 1=Diabetes
		Father's employment status at baseline	0=Unemployed; 1=Employed
		Father's highest education as binary outcome	1=High-school or lesser; 2=Post-secondary
		Father's self-reported ethnicity	1=NZ European; 2=Māori; 3=Pacific; 4=Asian; 5=Other
	**Child**	Sex	1=Female; 2=Male
		Grouping according to gestational age	1=Preterm; 2=Term/post-term
	**Household**	Area of residence	1=Urban; 2=Rural
		Family's household tenure	1=Home owners; 2=Tenants/others
		Type of baby feeding at 6 weeks	1=Breastmilk only; 2=Other
		New Zealand Index of Deprivation 2006 (stratified)	1 (least deprived); 2; 3; 4; 5 (most deprived)
**Continuous**	**Child**	Child's birth weight	–
		Child's birth weight *z*-score	–
		Change in weight *z*-score from birth	–
	**Mother**	Mother's age	–
		Mother's pre-pregnancy body mass index (BMI)	–
		Mother's pre-pregnancy weight	–
		Mother's birth weight	–
		Mother's height	–
	**Father**	Father's age	–
		Father's BMI at baseline	–
		Father's birth weight	–
		Father's height	–
		Father's weight at baseline	–
	**Household**	Number of other persons living in the house (other than the child)	–

Supplementary Table 2

Demographic information on the Growing Up in New Zealand (GUiNZ) participants included or excluded for model derivation and validation.

		Included	Excluded	p-value
**n**		2,444	3,270	
**Age (years)**		4.5 ± 0.1	4.6 ± 0.1	<0.001
**Sex ratio (males)**		1,289 (52.7%)	1,645 (50.3%)	0.069
**Birth weight *z*-score**		0.68 ± 1.00	0.75 ± 1.06	0.012
**Child BMI *z*-score**		0.68 ± 1.00	0.75 ± 1.06	0.012
**Child BMI status**	Underweight/normal weight	1,557 (63.7%)	1,794 (54.9%)	
	Overweight	498 (20.4%)	689 (21.1%)	
	Obesity	389 (15.9%)	787 (24.1%)	<0.001
**Number of other persons in household**		2 [2, 3]	3 [2, 4]	<0.001
**Maternal smoking during pregnancy**		113 (4.6%)	395 (14.4%)	<0.001
**Mother's ethnicity**	New Zealand European	1,725 (70.7%)	1,647 (50.6%)	
	Māori	170 (7.0%)	577 (17.7%)	
	Pacific	119 (4.9%)	589 (18.1%)	
	Asian	366 (15.0%)	395 (12.1%)	
	Other	61 (2.5%)	47 (1.4%)	<0.001
**Mother's education**	High-school or lesser	479 (19.6%)	1,164 (35.7%)	
	Post-secondary	663 (27.1%)	1,071 (32.9%)	
	University	1,301 (53.3%)	1,021 (31.4%)	<0.001
**Socioeconomic deprivation (NZDep2006)**	1 (least deprived)	533 (21.8%)	468 (14.9%)	
	2	549 (22.5%)	546 (17.4%)	
	3	483 (19.8%)	505 (16.1%)	
	4	488 (20.0%)	642 (20.5%)	
	5 (most deprived)	390 (16.0%)	970 (31.0%)	<0.001
**Mother's pre-pregnancy BMI (kg/m^2)**		24.89 ± 5.32	25.69 ± 6.16	<0.001
**Mother's pre-pregnancy BMI status**	Underweight/normal weight	1,518 (62.1%)	1,496 (57.1%)	
	Overweight	542 (22.2%)	598 (22.8%)	
	Obesity	384 (15.7%)	527 (20.1%)	<0.001
**Father's BMI (kg/m^2)**		27.28 ± 4.71	27.79 ± 5.34	0.004
**Father's BMI status**	Underweight/normal weight	827 (33.8%)	358 (30.1%)	
	Overweight	1,085 (44.4%)	533 (44.8%)	
	Obesity	532 (21.8%)	300 (25.2%)	0.022

BMI, body mass index; NZDep2006, New Zealand Index of Deprivation 2006.
The total number of participants only includes those children with available and valid anthropometric data.
The proportion of respondents in the Included or Excluded categories were compared using chi-squared tests.
Where appropriate, data are n (%) or means ± standard deviations (SD), except for Number of other persons in household where data are medians [quartile 1, quartile 3].
BMI status for children: underweight/normal weight BMI *z*-score <1.036; overweight BMI *z*-score ≥1.036 and <1.645; and obesity BMI *z*-score ≥1.645.
BMI status for mothers and fathers: underweight/normal weight BMI <25 kg/m^2; overweight ≥25 kg/m^2 and <30 kg/m^2; and obesity ≥30 kg/m^2.

Supplementary Table 3

Demographic information on the populations used for the derivation and validation of the prediction models for severe childhood obesity.

		GUiNZ derivation	GUiNZ validation	POI	PIF
**n**		2,408	1,027	513	523
**Age (years)**		4.5 ± 0.1	4.5 ± 0.1	5.0 ± 0.0	4.1 ± 0.2
**Sex (males)**		1,257 (52.2%)	538 (52.4%)	266 (51.9%)	262 (50.1%)
**Birth weight (kg)**		3.48 ± 0.58	3.48 ± 0.60	3.58 ± 0.48	3.59 ± 0.62
**Birth weight *z*-score**		0.70 ± 1.01	0.70 ± 1.06	0.59 ± 0.99	0.49 ± 1.14
**BMI *z*-score**		0.84 ± .1.10	0.85 ± 1.01	0.47 ± 0.90	1.77 ± 1.30
**Child BMI status**	Underweight/normal weight	1,479 (61.4%)	614 (59.8%)	398 (77.6%)	144 (27.5%)
	Overweight	491 (20.4%)	224 (21.8%)	74 (14.4%)	117 (22.4%)
	Obesity	438 (18.2%)	189 (18.4%)	41 (8.0%)	262 (50.1%)
	Severe obesity (≥99th %ile)	185 (7.7%)	69 (6.7%)	13 (2.5%)	145 (27.7%)
	Severe obesity (≥120% 95th %ile)	301 (12.5%)	125 (12.2%)	23 (4.5%)	199 (38.0%)
**Number of other persons in household**		2 [1, 3]	2 [1, 3]	3 [2, 3]	4 [3, 6]
**Maternal ethnicity**	New Zealand European	1,571 (65.3%)	656 (63.9%)	452 (88.1%)	-
	Māori	221 (9.2%)	108 (10.5%)	20 (3.9%)	-
	Pacific	208 (8.7%)	71 (6.9%)	5 (1.0%)	-
	Asian	357 (14.9%)	162 (15.8%)	24 (4.7%)	-
	Other	47 (2.0%)	30 (2.9%)	12 (2.3%)	-
**Maternal smoking during pregnancy**		163 (6.8%)	82 (8.0%)	42 (8.2%)	114 (21.8%)
**Maternal education**	High-school or lesser	572 (23.8%)	241 (23.5%)	96 (18.7%)	371 (70.9%)
	Post-secondary	704 (29.2%)	322 (31.4%)	67 (13.1%)	138 (26.4%)
	University	1,131 (47.0%)	463 (45.1%)	350 (68.2%)	14 (2.7%)
**NZDep2006**	1 (least deprived)	471 (19.6%)	216 (21.1%)	115 (22.7%)	-
	2	519 (21.6%)	207 (20.2%)	124 (24.5%)	-
	3	467 (19.4%)	189 (18.4%)	129 (25.5%)	-
	4	469 (19.5%)	214 (20.9%)	104 (20.6%)	-
	5 (most deprived)	481 (20.0%)	200 (19.5%)	34 (6.7%)	-
**Mother's pre-pregnancy BMI (kg/m^2)**		25.28 ± 5.66	25.22 ± 5.77	25.14 ± 5.10	37. 01 ± 7.65
**Mother's pre-pregnancy BMI status**	Underweight/normal weight	1,437 (59.7%)	618 (60.2%)	308 (60.0%)	24 (4.6%)
	Overweight	542 (22.5%)	220 (21.4%)	135 (26.3%)	65 (12.4%)
	Obesity	429 (17.8%)	189 (18.4%)	70 (13.6%)	434 (83.0%)
**Father's BMI (kg/m^2)**		27.29 ± 4.77	27.27 ± 4.58	27.09 ± 4.30	36.73 ± 6.59
**Father's BMI status**	Underweight/normal weight	585 (33.8%)	242 (33.9%)	131 (34.2%)	2 (1.5%)
	Overweight	762 (44.0%)	323 (45.3%)	171 (44.6%)	13 (9.6%)
	Obesity	384 (22.2%)	148 (20.8%)	81 (21.1%)	120 (88.9%)

BMI, body mass index; GUiNZ, Growing Up in New Zealand Study; NZDep2006, New Zealand Index of Deprivation 2006; PIF, Pacific Islands Families Study; POI, Prevention of Overweight in Infancy Study.

Where appropriate, data are n (%) or means ± standard deviations (SD), except for "Number of other persons in household" whose data are medians [quartile 1, quartile 3].

BMI status for children: underweight/normal weight BMI *z*-score <1.036; overweight BMI *z*-score ≥1.036 and <1.645; and obesity BMI *z*-score ≥1.645.

BMI status for mothers and fathers: underweight/normal weight BMI <25 kg/m^2; overweight ≥25 kg/m^2 and <30 kg/m^2; and obesity ≥30 kg/m^2.

Supplementary Table 4

Parameters for the severe childhood obesity prediction model derived from the Growing Up in New Zealand subset (severe childhood obesity defined as 120% of the 95th percentile).

	β	SEM	p-value	Odds ratio (95% CI)
**Birth weight *z*-score**	0.671	0.077	<0.0001	1.96 (1.68, 2.28)
**Maternal pre-pregnancy BMI (kg/m^2)**	0.079	0.010	<0.0001	1.08 (1.06, 1,10)
**Monthly weight gain from birth (per 0.1 *z*-score) ***	0.387	0.046	<0.0001	1.47 (1.35, 1.61)
**Maternal smoking during pregnancy**	0.831	0.209	<0.0001	2.30 (1.52, 3.46)
**Constant**	-4.695	0.286	<0.0001	-

BMI, body mass index; CI, confidence interval; SEM, standard error of the mean.

*Average monthly change in weight *z*-score from birth until the last recorded measurement between 6 and 12 months of age.

Supplementary Table 5

Parameters for the severe childhood obesity prediction model derived from the Growing Up in New Zealand subset (severe childhood obesity defined as ≥99th percentile).

	β	SEM	p-value	Odds ratio (95% CI)
**Birth weight *z*-score**	0.533	0.092	<0.0001	1.70 (1.42, 2.04)
**Maternal pre-pregnancy BMI (kg/m^2)**	0.091	0.012	<0.0001	1.10 (1.07, 1.12)
**Gestational age (week)**	0.065	0.048	0.171	1.07 (0.97, 1.17)
**Monthly weight gain from birth (per 0.1 *z*-score) ***	0.359	0.054	<0.0001	1.43 (1.29, 1.59)
**Maternal smoking during pregnancy**	1.125	0.23	<0.0001	3.08 (1.96, 4.83)
**Constant**	-8.099	1.901	<0.0001	–

BMI, body mass index; CI, confidence interval; SEM, standard error of the mean.

*Average monthly change in weight *z*-score from birth until the last recorded measurement between 6 and 12 months of age.

Supplementary Table 6

Accuracy and predictive capacity of a prediction model for severe childhood obesity for New Zealand among the derivation and validation cohorts (severe childhood obesity defined as 120% of the 95th percentile).

		PROBABILITY PERCENTILE THRESHOLD															
**MODEL**	**PARAMETER**	**20**	**25**	**30**	**35**	**40**	**45**	**50**	**55**	**60**	**65**	**70**	**75**	**80**	**85**	**90**	**95**
**GUiNZ derivation model**	True positives (n)	289	278	273	267	257	249	241	230	217	204	192	175	145	121	92	58
**n = 2,408**	True negatives (n)	470	579	695	809	919	1032	1144	1253	1361	1468	1576	1680	1770	1866	1958	2044
**AUROC = 0.75 (0.72, 0.78)**	False positives (n)	1637	1528	1412	1298	1188	1075	963	854	746	639	531	427	337	241	149	63
	False negatives (n)	12	23	28	34	44	52	60	71	84	97	109	126	156	180	209	243
	Sensitivity (%)	96.0	92.4	90.7	88.7	85.4	82.7	80.1	76.4	72.1	67.8	63.8	58.1	48.2	40.2	30.6	19.3
	Specificity (%)	22.3	27.5	33.0	38.4	43.6	49.0	54.3	59.5	64.6	69.7	74.8	79.7	84.0	88.6	92.9	97.0
	PPV (%)	15.0	15.4	16.2	17.1	17.8	18.8	20.0	21.2	22.5	24.2	26.6	29.1	30.1	33.4	38.2	47.9
	NPV (%)	97.5	96.2	96.1	96.0	95.4	95.2	95.0	94.6	94.2	93.8	93.5	93.0	91.9	91.2	90.4	89.4
**GUiNZ internal validation model**	True positives (n)	118	116	113	109	104	100	97	92	87	85	81	73	66	56	45	25
**n = 1,027**	True negatives (n)	187	238	290	338	390	429	471	517	568	617	660	698	734	784	829	878
**AUROC = 0.74 (0.69, 0.79)**	False positives (n)	715	664	612	564	512	473	431	385	334	285	242	204	168	118	73	24
	False negatives (n)	7	9	12	16	21	25	28	33	38	40	44	52	59	69	80	100
	Sensitivity (%)	94.4	92.8	90.4	87.2	83.2	80.0	77.6	73.6	69.6	68.0	64.8	58.4	52.8	44.8	36.0	20.0
	Specificity (%)	20.7	26.4	32.2	37.5	43.2	47.6	52.2	57.3	63.0	68.4	73.2	77.4	81.4	86.9	91.9	97.3
	PPV (%)	14.2	14.9	15.6	16.2	16.9	17.5	18.4	19.3	20.7	23.0	25.1	26.4	28.2	32.2	38.1	51.0
	NPV (%)	96.4	96.4	96.0	95.5	94.9	94.5	94.4	94.0	93.7	93.9	93.8	93.1	92.6	91.9	91.2	89.8
**POI external validation model**	True positives (n)	21	21	21	21	19	19	19	19	19	17	13	12	11	8	7	3
**n = 513**	True negatives (n)	104	121	147	174	201	228	256	289	315	355	373	393	410	438	456	475
**AUROC = 0.77 (0.66, 0.88)**	False positives (n)	386	369	343	316	289	262	234	201	175	135	117	97	80	52	34	15
	False negatives (n)	2	2	2	2	4	4	4	4	4	6	10	11	12	15	16	20
	Sensitivity (%)	91.3	91.3	91.3	91.3	82.6	82.6	82.6	82.6	82.6	73.9	56.5	52.2	47.8	34.8	30.4	13.0
	Specificity (%)	21.2	24.7	30.0	35.5	41.0	46.5	52.2	59.0	64.3	72.4	76.1	80.2	83.7	89.4	93.1	96.9
	PPV (%)	5.2	5.4	5.8	6.2	6.2	6.8	7.5	8.6	9.8	11.2	10.0	11.0	12.1	13.3	17.1	16.7
	NPV (%)	98.1	98.4	98.7	98.9	98.0	98.3	98.5	98.6	98.7	98.3	97.4	97.3	97.2	96.7	96.6	96.0

		PROBABILITY PERCENTILE THRESHOLD															
**MODEL**	**PARAMETER**	**20**	**25**	**30**	**35**	**40**	**45**	**50**	**55**	**60**	**65**	**70**	**75**	**80**	**85**	**90**	**95**
**PIF external validation model**	True positives (n)	197	197	194	194	192	192	191	186	183	180	175	165	157	144	107	76
**n = 523**	True negatives (n)	6	8	10	17	25	27	33	53	63	76	90	107	132	165	204	263
**AUROC = 0.69 (0.64, 0.74)**	False positives (n)	318	316	314	307	299	297	291	271	261	248	234	217	192	159	120	61
	False negatives (n)	2	2	5	5	7	7	8	13	16	19	24	34	42	55	92	123
	Sensitivity (%)	99.0	99.0	97.5	97.5	96.5	96.5	96.0	93.5	92.0	90.5	87.9	82.9	78.9	72.4	53.8	38.2
	Specificity (%)	1.9	2.5	3.1	5.2	7.7	8.3	10.2	16.4	19.4	23.5	27.8	33.0	40.7	50.9	63.0	81.2
	PPV (%)	38.3	38.4	38.2	38.7	39.1	39.3	39.6	40.7	41.2	42.1	42.8	43.2	45.0	47.5	47.1	55.5
	NPV (%)	75.0	80.0	66.7	77.3	78.1	79.4	80.5	80.3	79.7	80.0	78.9	75.9	75.9	75.0	68.9	68.1

AUROC, area under the receiver operating characteristic curve; GUiNZ, Growing Up in New Zealand Study; NPV, negative predictive value; PIF, Pacific Islands Families Study; POI, Prevention of Overweight in Infancy Study; PPV, positive predictive value.

Supplementary Table 7

Accuracy and predictive capacity of a prediction model for childhood obesity for New Zealand among the derivation and validation cohorts (severe childhood obesity defined as ≥99th percentile).

		PROBABILITY PERCENTILE THRESHOLD															
**MODEL**	**PARAMETER**	**20**	**25**	**30**	**35**	**40**	**45**	**50**	**55**	**60**	**65**	**70**	**75**	**80**	**85**	**90**	**95**
**GUiNZ derivation model**	True positives (n)	173	171	168	163	159	155	147	143	140	135	129	120	105	90	71	40
**n = 2,408**	True negatives (n)	470	588	706	821	937	1054	1166	1282	1400	1515	1629	1741	1846	1951	2053	2142
**AUROC = 0.76 (0.72, 0.80)**	False positives (n)	1753	1635	1517	1402	1286	1169	1057	941	823	708	594	482	377	272	170	81
	False negatives (n)	12	14	17	22	26	30	38	42	45	50	56	65	80	95	114	145
	Sensitivity (%)	93.5	92.4	90.8	88.1	85.9	83.8	79.5	77.3	75.7	73.0	69.7	64.9	56.8	48.6	38.4	21.6
	Specificity (%)	21.1	26.5	31.8	36.9	42.2	47.4	52.5	57.7	63.0	68.2	73.3	78.3	83.0	87.8	92.4	96.4
	PPV (%)	9.0	9.5	10.0	10.4	11.0	11.7	12.2	13.2	14.5	16.0	17.8	19.9	21.8	24.9	29.5	33.1
	NPV (%)	97.5	97.7	97.6	97.4	97.3	97.2	96.8	96.8	96.9	96.8	96.7	96.4	95.8	95.4	94.7	93.7
**GUiNZ internal validation model**	True positives (n)	65	64	61	58	56	55	54	52	51	47	46	44	41	36	28	19
**n = 713**	True negatives (n)	192	246	305	350	399	443	487	541	596	651	691	731	770	821	868	922
**AUROC = 0.73 (0.68, 0.78)**	False positives (n)	766	712	653	608	559	515	471	417	362	307	267	227	188	137	90	36
	False negatives (n)	4	5	8	11	13	14	15	17	18	22	23	25	28	33	41	50
	Sensitivity (%)	94.2	92.8	88.4	84.1	81.2	79.7	78.3	75.4	73.9	68.1	66.7	63.8	59.4	52.2	40.6	27.5
	Specificity (%)	20.0	25.7	31.8	36.5	41.6	46.2	50.8	56.5	62.2	68.0	72.1	76.3	80.4	85.7	90.6	96.2
	PPV (%)	7.8	8.2	8.5	8.7	9.1	9.6	10.3	11.1	12.3	13.3	14.7	16.2	17.9	20.8	23.7	34.5
	NPV (%)	98.0	98.0	97.4	97.0	96.8	96.9	97.0	97.0	97.1	96.7	96.8	96.7	96.5	96.1	95.5	94.9
**POI external validation model**	True positives (n)	30	29	27	26	25	22	22	18	15	13	11	8	7	6	4	0
**n = 513**	True negatives (n)	76	102	125	149	180	199	217	237	265	308	337	363	383	409	438	460
**AUROC = 0.78 (0.62, 0.95)**	False positives (n)	404	378	355	331	300	281	263	243	215	172	143	117	97	71	42	20
	False negatives (n)	3	4	6	7	8	11	11	15	18	20	22	25	26	27	29	33
	Sensitivity (%)	90.9	87.9	81.8	78.8	75.8	66.7	66.7	54.5	45.5	39.4	33.3	24.2	21.2	18.2	12.1	0.0
	Specificity (%)	15.8	21.3	26.0	31.0	37.5	41.5	45.2	49.4	55.2	64.2	70.2	75.6	79.8	85.2	91.3	95.8
	PPV (%)	6.9	7.1	7.1	7.3	7.7	7.3	7.7	6.9	6.5	7.0	7.1	6.4	6.7	7.8	8.7	0.0
	NPV (%)	96.2	96.2	95.4	95.5	95.7	94.8	95.2	94.0	93.6	93.9	93.9	93.6	93.6	93.8	93.8	93.3

		PROBABILITY PERCENTILE THRESHOLD															
**MODEL**	**PARAMETER**	**20**	**25**	**30**	**35**	**40**	**45**	**50**	**55**	**60**	**65**	**70**	**75**	**80**	**85**	**90**	**95**
**PIF external validation model**	True positives (n)	59	57	50	44	40	34	31	26	26	18	14	11	9	6	4	2
**n = 523**	True negatives (n)	295	308	321	328	335	343	344	347	354	359	363	364	369	372	375	377
**AUROC = 0.71 (0.66, 0.76)**	False positives (n)	83	70	57	50	43	35	34	31	24	19	15	14	9	6	3	1
	False negatives (n)	86	88	95	101	105	111	114	119	119	127	131	134	136	139	141	143
	Sensitivity (%)	40.7	39.3	34.5	30.3	27.6	23.4	21.4	17.9	17.9	12.4	9.7	7.6	6.2	4.1	2.8	1.4
	Specificity (%)	78.0	81.5	84.9	86.8	88.6	90.7	91.0	91.8	93.7	95.0	96.0	96.3	97.6	98.4	99.2	99.7
	PPV (%)	41.5	44.9	46.7	46.8	48.2	49.3	47.7	45.6	52.0	48.6	48.3	44.0	50.0	50.0	57.1	66.7
	NPV (%)	77.4	77.8	77.2	76.5	76.1	75.6	75.1	74.5	74.8	73.9	73.5	73.1	73.1	72.8	72.7	72.5

AUROC, area under the receiver operating characteristic curve; GUiNZ, Growing Up in New Zealand Study; NPV, negative predictive value; PIF, Pacific Islands Families Study; POI, Prevention of Overweight in Infancy Study; PPV, positive predictive value.

Supplementary Figure 1

Flow of participants from Growing Up in New Zealand who were included in model derivation and validation.

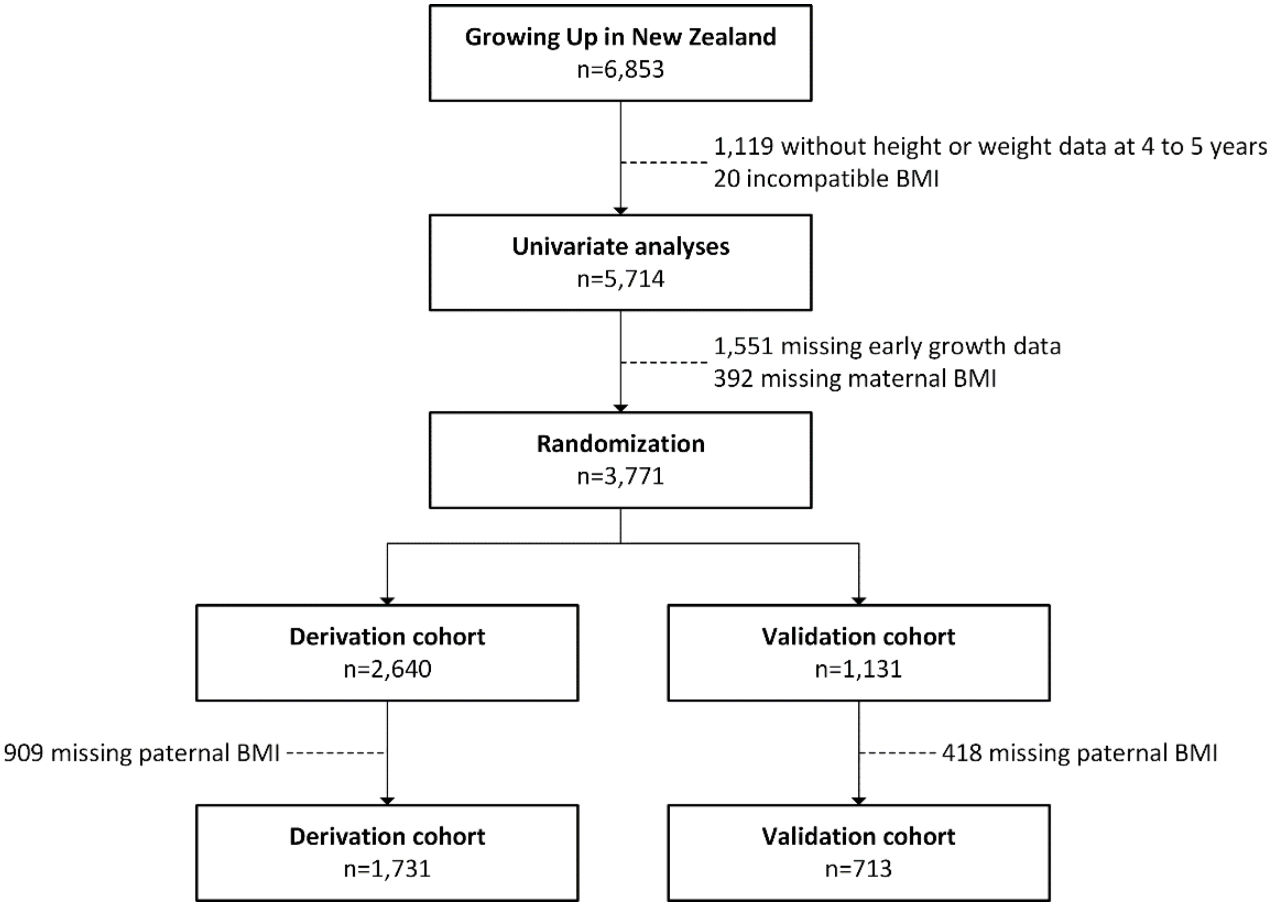

Supplement: Supplementary file 1 — Supplementary Information [file 41598_2021_85557_MOESM1_ESM.pdf]
